# Supplementary material for: Pentoxifylline Enhances the Effects of Doxorubicin and Bleomycin on Apoptosis, Caspase Activity, and Cell Cycle While Reducing Proliferation and Senescence in Hodgkin’s Disease Cell Line
Source: Curr Issues Mol Biol. 2025 Jul 28;47(8):593. doi: 10.3390/cimb47080593 (PMC12384627; doi:10.3390/cimb47080593)
Supplement: Supplementary file 1 [file cimb-47-00593-s001.zip › Figure_S1.pdf]

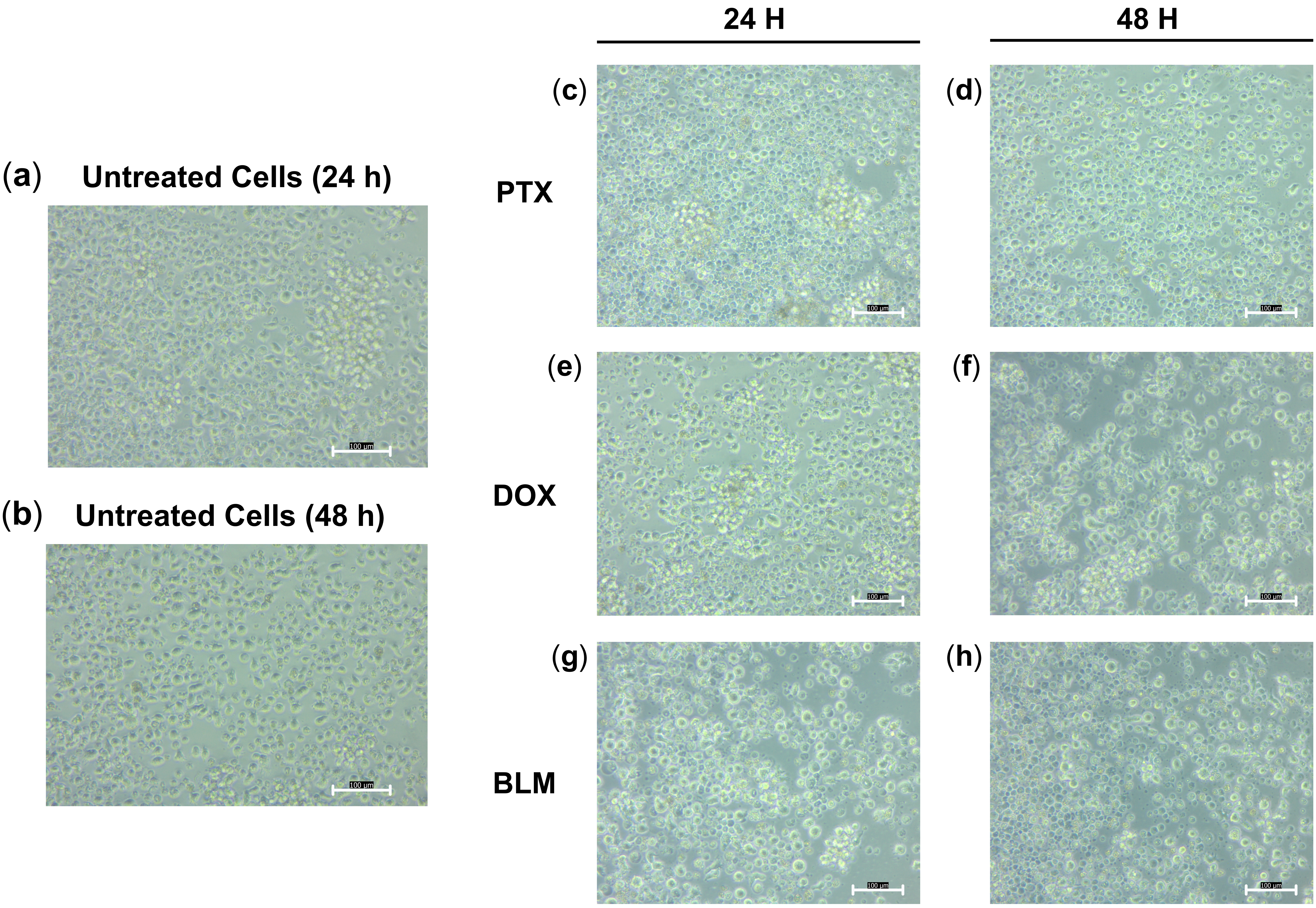

Figure S1. Cell Morphology of Hs-445 HL Cells Treated with PTX, DOX, and BLM. (**a-b)** Baseline morphology of untreated Hs-445 HL cells at 24 and 48 h (control group). (**c-d**) Morphology of Hs-445 cells treated with PTX at 8 mM for 24 and 48 h, respectively. (**e-f**) Morphology of Hs-445 cells treated with DOX at 1 μM for 24 and 48 h, respectively. (**g-h**) Morphology of Hs-445 cells treated with BLM at 100 mU for 24 and 48 h, respectively. Images were captured using a ZEISS Primovert inverted microscope with a 20X objective (scale bar = 100 μM). BLM: Bleomycin; DOX: Doxorubicin; PTX: Pentoxifylline.
