# Supplementary material for: Pentoxifylline Enhances the Effects of Doxorubicin and Bleomycin on Apoptosis, Caspase Activity, and Cell Cycle While Reducing Proliferation and Senescence in Hodgkin’s Disease Cell Line
Source: Curr Issues Mol Biol. 2025 Jul 28;47(8):593. doi: 10.3390/cimb47080593 (PMC12384627; doi:10.3390/cimb47080593)
Supplement: Supplementary file 1 [file cimb-47-00593-s001.zip › Figure_S2.pdf]

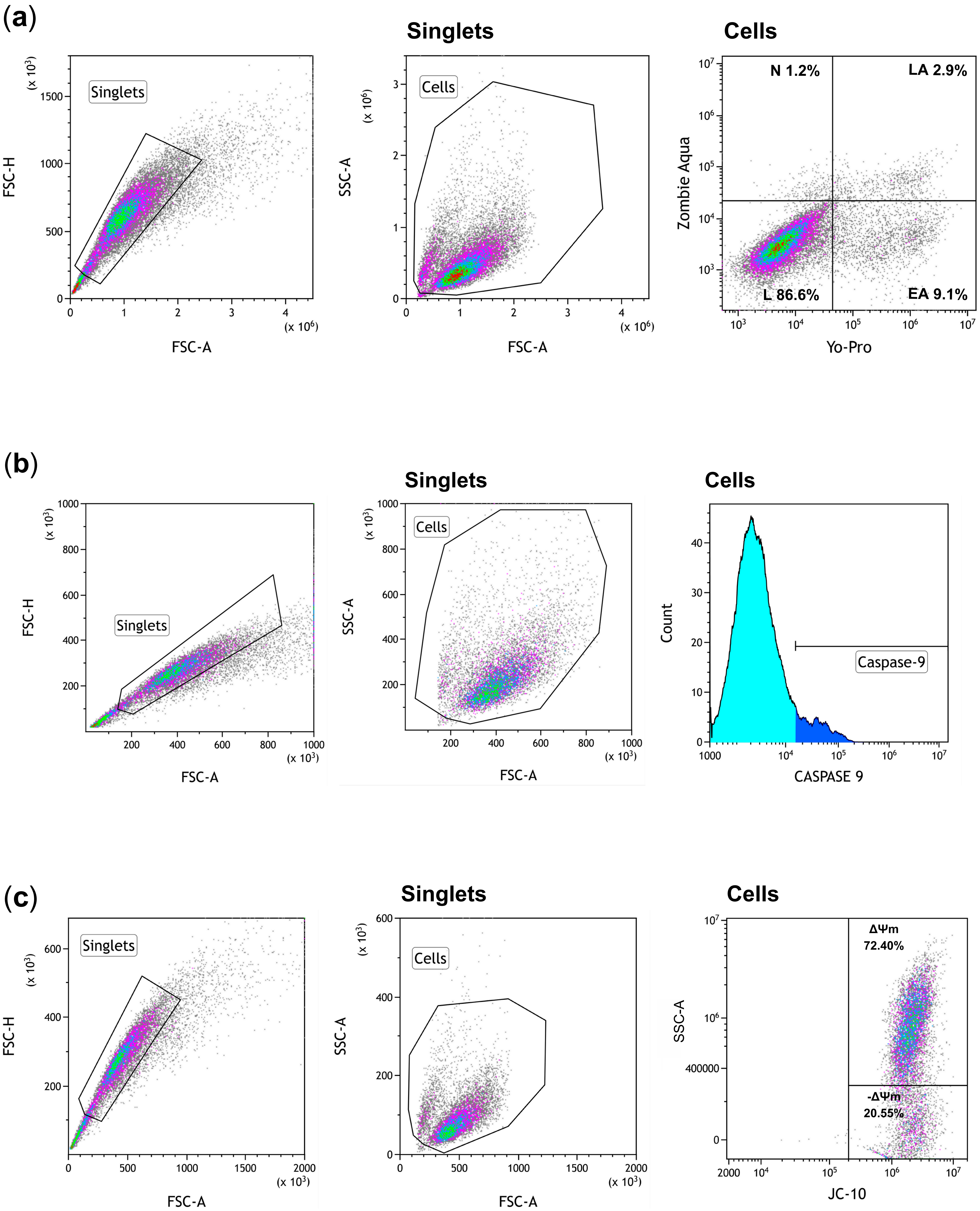
(a)
Singlets
Cells
Singlets
FSC-H
(x 10^3)
FSC-A
(x 10^6)
Cells
SSC-A
(x 10^6)
FSC-A
(x 10^6)
N 1.2%
LA 2.9%
L 86.6%
EA 9.1%
Zombie Aqua
Yo-Pro
(b)
Singlets
Cells
Singlets
FSC-H
(x 10^3)
FSC-A
(x 10^3)
Cells
SSC-A
(x 10^3)
FSC-A
(x 10^3)
Caspase-9
Count
CASPASE 9
(c)
Singlets
Cells
Singlets
FSC-H
(x 10^3)
FSC-A
(x 10^3)
Cells
SSC-A
(x 10^3)
FSC-A
(x 10^3)
ΔΨm
72.40%
-ΔΨm
20.55%
SSC-A
JC-10

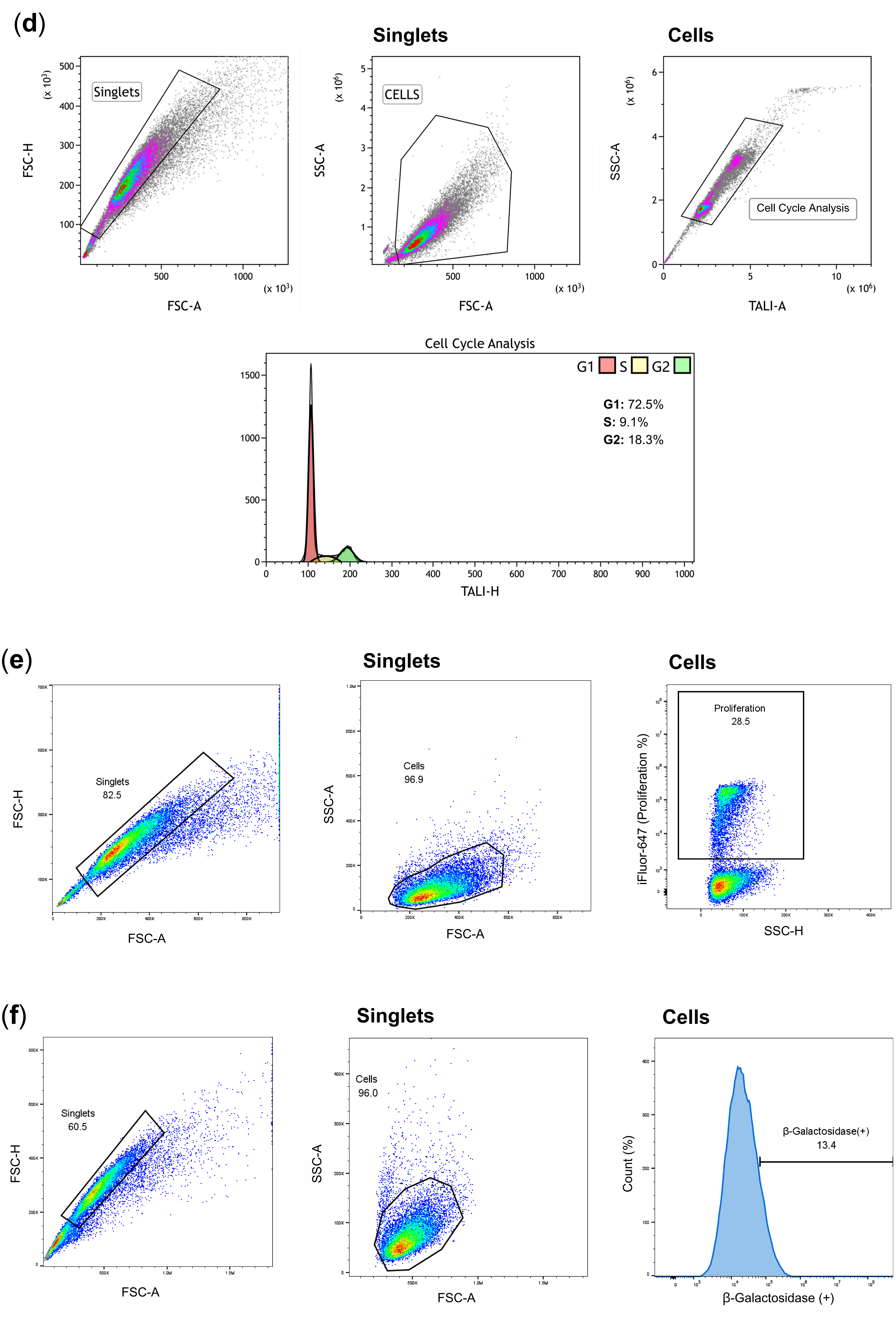

(d)
Singlets
Cells
Singlets
FSC-H
FSC-A
CELLS
SSC-A
Cell Cycle Analysis
TALI-A
Cell Cycle Analysis
G1 S G2
G1: 72.5%
S: 9.1%
G2: 18.3%
TALI-H
(e)
Singlets
Cells
Singlets
82.5
Cells
96.9
Proliferation
28.5
iFluor-647 (Proliferation %)
SSC-H
(f)
Singlets
Cells
Singlets
60.5
Cells
96.0
β-Galactosidase(+)
13.4
Count (%)
β-Galactosidase (+)

Figure S2. Flow cytometry gating strategies. (**a**) Cell apoptosis analysis using Zombie Aqua and Yo-Pro viability dyes. (**b**) Assessment of caspase-3, -8, and -9 activity with FITC-DEVD-FMK, FITC-IETD-FMK, and FITC-LEHD-FMK, respectively. (**c**) Evaluation of mitochondrial membrane potential using JC-10 dye. (**d**) Cell cycle analysis was performed with propidium iodide, included in the Tali reagent used in this assay. (**e**) Cell proliferation assay based on the incorporation of the thymidine analog EdU conjugated to iFluor-647 dye. (**f**) Detection of β-galactosidase for evaluating cell senescence.
