# Supplementary material for: Pentoxifylline Enhances the Effects of Doxorubicin and Bleomycin on Apoptosis, Caspase Activity, and Cell Cycle While Reducing Proliferation and Senescence in Hodgkin’s Disease Cell Line
Source: Curr Issues Mol Biol. 2025 Jul 28;47(8):593. doi: 10.3390/cimb47080593 (PMC12384627; doi:10.3390/cimb47080593)
Supplement: Supplementary file 1 [file cimb-47-00593-s001.zip › Table_S2.pdf]

Table S2. Chemical Structures of Carboplatin, Docetaxel, Doxorubicin, and Simvastatin.

Name	Molecular Formula	Chemical Structure	PubChem CID
Carboplatin	$C_6H_{12}N_2O_4Pt$	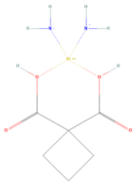	426756
Docetaxel	$C_{45}H_{55}NO_{15}$	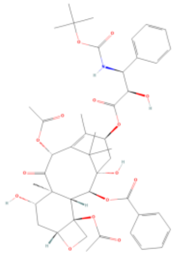	147895
Doxorubicin	$C_{27}H_{29}NO_{11B}$	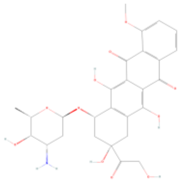	31703
Simvastatin	$C_{13}H_{18}N_4O_3$	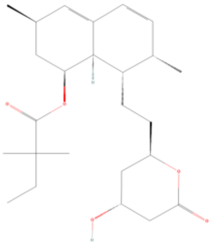	54454

CID: Compound Identifier
